# 

**Published:** 1864-03

**Authors:** 


### CHICAGO MEDICAL JOURNAL.

**Terms, $2.00 per Annum.**

### CONTENTS OF THE MARCH NUMBER.

PAGE.

ORIGINAL COMMUNICATIONS.

Ligature of the Common Iliac Artery. By Daniel Brainard, M. D., Professor of Surgery in Rush Medical College........................................ 97

Treatment of Permanent Stricture of the Urethra by Sudden Dilitation. By Wm. B. Slayter, M. D., of Chicago, Graduate Royal College of Physicians of England, etc ........................................ 99

On the Pathogenetic Effects of Malaria. By W. G. Munson, M. D...... 102

Clinical Lectures on Diseases of the Eye. By E. L. Holmes, M. D., of Chicago, Lecturer on Diseases of the Eye and Ear in Rush Medical College, etc.—The Cornea—Anatomy and Pathology.................. 114

SELECTED.

On the Presence of Air in the Veins as a Cause of Death. By Jas. Sumner Greene, M. D., of Dorchester, Mass......... ........................ 120

Editorial Correspondence........................................ 136

Book Notices and Reviews........................................ 141

